# Red blood cell transfusions post diagnosis of necrotizing enterocolitis and the deterioration of necrotizing enterocolitis in full-term and near-term infants: a propensity score adjustment retrospective cohort study

**DOI:** 10.1186/s12887-022-03276-4

**Published:** 2022-04-15

**Authors:** Lijuan Luo, Xingling Liu, Huan Yu, Mei Luo, Wen Jia, Wenbin Dong, Xiaoping Lei

**Affiliations:** 1 Division of Neonatology, Department of Pediatrics, The Affiliated Hospital of Southwest Medical University, 8 Kangcheng Road, Luzhou, Sichuan China; 2 Department of Perinatology, The Affiliated Hospital of Southwest Medical University, Luzhou, Sichuan China; 3 Sichuan Clinical Research Center for Birth Defects, Luzhou, Sichuan China

**Keywords:** Necrotizing enterocolitis, Red blood transfusion, Deterioration, Propensity score, Infants

## Abstract

**Background:**

Necrotizing enterocolitis (NEC) is one of serious gastrointestinal inflammatory diseases in newborn infants, with a high morbidity and mortality. Red blood cell transfusion (RBCT) plays a controversial and doubtful role in the treatment of NEC. In present study, we aim to analyze the association between RBCT and the deterioration of NEC.

**Methods:**

This was a retrospective cohort study of near-term and full-term infants with a confirmed diagnosis of Bell’s stage II NEC between Jan 1, 2010 and Jan 31, 2020. The maternal and infant baseline characteristics, treatment information and laboratory test for each case were collected. The eligible subjects were divided into two groups based on receiving RBCT post NEC diagnosis or not. The propensity score was used to eliminate potential bias and baseline differences. A multivariate logistic regression model was used to adjust the propensity score and calculate the odds ratio (OR) and 95% confidential interval (CI) of RBCT for the deterioration of NEC.

**Results:**

A total of 242 infants were included in this study, 60 infants had a history of RBCT post NEC diagnosis, and 40 infants deteriorated from Bell’s stage II to stage III. By adjusting the propensity score, RBCT post NEC diagnosis was associated with an increased risk for NEC deteriorating from stage II to III (adjusted OR 6.06, 95%CI 2.94–12.50, *P* = 0.000).

**Conclusions:**

NEC infants who required RBCT post NEC diagnosis were more likely to deteriorate from stage II to III in full-term and near-term infants.

**Supplementary Information:**

The online version contains supplementary material available at 10.1186/s12887-022-03276-4.

## Introduction

Necrotizing enterocolitis (NEC) remains one of the common and serious gastrointestinal inflammatory diseases in newborn infants, with an estimated incidence of 1–5 per 1000 live births [1], and mortality rates ranging from 20 to 30% among neonates requiring surgery [2]. The spectrum of NEC ranges from a slowly evolving and benign form to a more serious or advanced form. According to the staging system proposed by Bell in the 1970s originally, modified by Walsh and Kliegman subsequently [3], NEC was classified into 3 stages. Stage III NEC cases had a higher rate of mortality, higher cost of hospitalization, and more significant sequelae than those less severe NEC cases [4]. For infants with stage III NEC, some of them have stage III documented within the first 24 to 48 h after the onset of NEC, but most of them were progressed from less severe conditions, such as stage I or II [3]. Therefore, if it were somehow possible to prevent stage I or II NEC from progressing to stage III, the prognosis of NEC would probably be improved. However, little information is available to explain why some less severe NEC episodes are managed successfully with medical intensive care, while certain cases deteriorate to stage III. Previous studies had found that broad-spectrum antibiotics plus anaerobic antimicrobial therapy [5, 6] and probiotics [7] could not prevent the deterioration of NEC. Thus, it is a priority to explore the risk factors for the deterioration of NEC.

Red blood cell transfusion (RBCT), a common intervention in neonatal intensive care units, plays a controversial and doubtful role in the treatment of NEC. Several observational studies demonstrated that a positive association was observed between RBCT and the subsequent development of NEC [8, 9]. However, no association [10, 11], even a negative association [12, 13] between RBCT and the subsequent development of NEC were observed in other studies. In clinical practice, RBCT was an alternative strategy for treating evolving NEC infants with anemia. Beena G. et al. found that infants were more likely to receive RBCT after diagnosis of NEC than before, and approximately 90% premature infants with NEC received RBCT after diagnosis of NEC [12]. What a effect does RBCT have on the deterioration of NEC is unclear. The main literature about the associations between NEC and RBCT focused on premature infants [10, 12], and premature infants presented with different patterns of disease and have different outcomes compared with full term infant [14]. It suggests NEC in preterm infants may be a different clinical entity from the full term infants. In addition, the previous literature [10, 12] about the associations between NEC and RBCT focused on association between RBCT and the onset of NEC instead of the deterioration of NEC. Considering most infants with stage I NEC are not really NEC, but much more probability of feeding intolerance, to minimize ascertainment bias from feeding intolerance, we focused on the near-term and full term infants (gestational age ≥ 34 weeks and ≤ 42 weeks) with stage II NEC to explore the association between RBCT and the deterioration of NEC in the present study.

## Methods

### Data collection

The Affiliated Hospital of Southwest Medical University is one of the tertiary referral centers located in southwest of China. And the neonatal intensive care unit (NICU) received almost all the high risk newborns from the neighborhood hospitals. As a retrospective cohort study, the data were extracted from the hospital information system and used anonymously. Ethical approval and written informed consent were granted an exemption for using the anonymous clinical data from ethics committee of the Affiliated Hospital of Southwest Medical University. All methods were carried out in accordance with relevant guidelines and regulations.

In the present study, the eligible study subjects were the near-term and full-term infants with a confirmed diagnosis of Bell’s stage II NEC [3] between Jan 1, 2010 and Jan 31, 2020. The maternal and infant baseline characteristics were collected from the HIS. The extracted maternal information included pregnancy-induced hypertension, maternal diabetes, antenatal corticosteroids use, amniotic fluid contamination, rupture of membrane > 18 h, mode of delivery, and the extracted infant baseline characteristics included gestational age, birth weight, gender, multiple gestation, feeding method before hospitalization, the age of NEC onset, the age of NEC diagnosis, congenital heart disease, pathoglycemia, sepsis, coagulopathy, scleredema neonatorum, intracranial hemorrhage, metabolic acidosis, hemolytic disease of newborn, asphyxia, liver dysfunction, renal dysfunction, etc.

The infant white blood cell count, platelet count, C-reactive protein (CRP), hematocrit (Hct) and abdominal radiographic and ultrasonic reports during hospitalization were reviewed. The treatment information was also collected, including days for first cessation of enteral feeding, days for first naso-gastric suction, days for antibiotics (broad spectrum antibiotics and anaerobic antimicrobial therapy), blood products transfusion (red blood cell, fresh frozen plasma, platelet, cryoprecipitate, albumin, and intravenous immunoglobulin), probiotics, vasoactive agents (dapamin, dobutamine, adrenaline, etc) and mechanical ventilation.

In order to minimize the bias, the diagnosis and stage of each NEC case were reviewed by a senior neonatologist who was blinded in the purpose by checking all the clinical records and abdominal radiographs (images and reports) according to the established criteria [3]. All the NEC infants received the similar basic treatment following the guideline, including fasting, parenteral antibiotics, gastrointestinal decompression (naso-gastricsuction, and anal tube), intravenous nutrition, and intensive care therapy (cardio-respiratory support and blood products transfusion) if necessary.

In general, the decision for RBCT was made following the guidelines for transfusion of RBCs in patients less than 4 months of age (seen in supplement 1.) [15]. However, neonatologists were also allowed to exercise discretion and order a RBCT if an infant deteriorated unexpectedly or was judged to be more likely to benefit from RBCT even if the recommended guideline had not been met. Each RBCT consisted of 10–20 ml/kg and was given over 2–4 h. Feedings were not routinely withheld during RBCT.

The age of NEC onset was defined as the day on which at least 1 of the following signs or symptoms occurred: prefeeding gastric residuals, emesis, abdominal distension, or bloody stool. The age of NEC diagnosis was defined as the day on which the abdominal X-ray or the ultrasound result fit the diagnostic criteria for stage II NEC. The deterioration of NEC was defined as NEC case progressing from bell stage II to stage III. Gestational age was confirmed by the first trimester ultrasound or the maternal last menstrual period. Congenital heart disease was diagnosed by routine echocardiography screening. Tiny-to-small PDA or patent foramen ovale were not included in congenital heart disease in the present study, for the ubiquity of small ductuses in the neonatal population and lacking of evidence of reduced systemic perfusion from the ductal steal leading to insufficient mesenteric circulation and intestinal ischemia [16]. Sepsis included culture-positive sepsis and clinical diagnosed sepsis. The white blood cell count, platelet count and CRP which tested within 24 h of NEC diagnosis were considered as the baseline values.

### Statistics

A chi-square test was used to compare the categorical variables between the group with and without RBCT post diagnosis of NEC. For continuous variables, an independent 2-tailed t-test and Mann-Whitney U tests were used for normally distributed variables and skewed distributed variables between the two groups, respectively.

The propensity score was used to estimate the treatment assignment probability [17]. It is a popular approach for eliminating potential bias and baseline differences to allow for a more reasonable comparison between the study and the control groups in observational studies, and to some extend it can prevent sparse data bias [17, 18]. In this retrospective observational study, many factors, including gestational age, birth weight, et al. could affect the progression of NEC [4], and the RBCT in NEC cases was not ordered by a randomization. A propensity score was calculated by a logistic regression model for each patient, based on the potential influential factors of RBCT [17, 19], including the maternal and infant baseline characteristics, complications, the baseline value of blood routine test and CRP, and the treatment strategies. Then, another logistic regression model was used to calculate the odds ratio (OR) and 95% confidential interval (CI) of RBCT for the deterioration of NEC, after adjusting the propensity score. All statistics were processed with SPSS 25.0 (SPSS Inc., Chicago, IL).

## Results

**Fig. 1:**
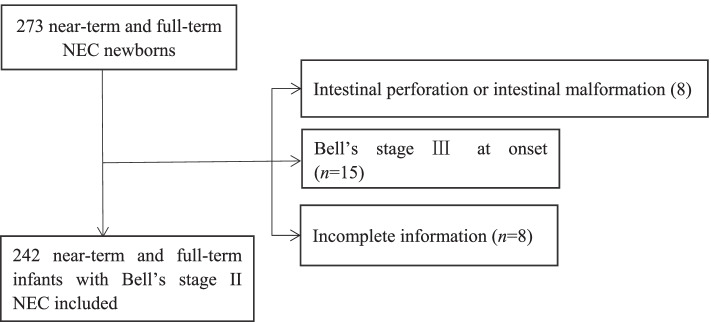
Flow Chart in the Selection of infants with Bell’s stage II NEC

Of the 273 eligible near-term and full-term newborns with NEC, 8 infants with intestinal perforation or intestinal malformation (aproctia, intestinal atresia, Hirschsprung’s disease), 15 infants with Bell’s stage III NEC at the onset of the disease and 8 infants with incomplete information were excluded. Finally, 242 infants with Bell’s stage II NEC met the inclusion criterion (seen in Fig. 1).

Overall, of the 242 infants enrolled, 60 (24.7%) infants had a history of RBCT post NEC diagnosis, and 40 (16.5%) infants deteriorated from Bell’s stage II to stage III. The demographic features of all infants were shown in Table 1. The infants with RBCT post NEC had a higher rate of caesarean delivery (78.3% vs 62.1%, *P* < 0.05), an elder of age at diagnosis of NEC (10.5 (3.25–26.75) vs 8 (2–16), *P* < 0.05), and a lower birth weight (2763 g ± 614 g vs 2989 g ± 580 g, *P* < 0.05).

**Table 1:** The demographic characteristics of infants with or without red blood cell transfusion post diagnosis of necrotizing enterocolitis

Variable	Red blood cell transfusion post diagnosis of necrotizing enterocolitis		
	With (*n* = 60)	Without (*n* = 182)	*P*
Pregnancy induced hypertension, % (n)	10.0 (6)	3.8 (7)	0.133
Maternal diabetes, % (n)	1.7 (1)	3.3 (6)	0.834
Antenatal use of corticosteroids, % (n)	1.7 (1)	0	0.248
Amniotic fluid contamination, % (n)	11.7 (7)	9.3 (17)	0.601
Rupture of membranes > 18 h, % (n)	1.7 (1)	4.4 (8)	0.565
Caesarean delivery, % (n)	78.3 (47)	62.1 (113)	0.021
Breast fed at home, % (n)	21.7 (13)	21.4 (39)	0.803
Male, % (n)	66.7 (40)	61.5 (112)	0.476
Multiple gestations, % (n)	10.0(6)	5.5(10)	0.358
Small for gestational age, % (n)	63.3 (38)	74.2 (135)	0.107
Age of NEC onset [days, median (IQR)]	6 (2–17.5)	5 (1–11)	0.088
Age of NEC diagnosis [days, median (IQR)]	10.5 (3.25–26.75)	8 (2–16)	0.033
Birth weight (grams, mean ± SD)	2763 ± 614	2989 ± 580	0.011
Gestational age [weeks, median (IQR)]	38.1 (36.1–39.5)	38.9 (37.3–39.7)	0.076

*SD* Standard deviation, *IQR* Interquartile range

As shown in Table 2, the infants with RBCT post NEC had a higher risk in sepsis (55.0% vs 21.4%, *P* = 0.000), and no significant difference was observed in other complications.

**Table 2:** The complications of infants with or without red blood cell transfusion post diagnosis of necrotizing enterocolitis

Complications	Red blood cell transfusion post diagnosis of necrotizing enterocolitis		
	With (*n* = 60)	Without (*n* = 182)	*P*
Congenital heart disease^a^, %(n)	38.3 (23)	36.3 (66)	0.773
Pathoglycemia, %(n)	10.0 (6)	6.6 (12)	0.556
Sepsis, %(n)	55.0 (33)	21.4 (39)	0.000
Coagulopathy, %(n)	23.3 (14)	36.8 (67)	0.055
Scleredema neonatorum, %(n)	5.0 (3)	5.5 (10)	1.0
Intracranial hemorrhage, %(n)	13.3 (8)	7.1 (13)	0.14
Metabolic acidosis, %(n)	8.3 (5)	2.7 (5)	0.131
Hemolytic disease of newborn, %(n)	16.7(10)	14.8 (27)	0.732
Asphyxia, %(n)	1.7 (1)	4.4 (8)	0.565
Liver dysfunction, %(n)	11.7 (7)	3.8 (7)	0.053
Renal dysfunction, %(n)	16.7 (10)	9.3 (17)	0.118

Congenital heart disease^a^: Tiny-to-small PDA or patent foramen ovale were not included

The infants with RBCT post NEC diagnosis had the a higher rate in receiving transfusion of fresh frozen plasma (25% vs 2.2%, *P* = 0.000), platelet (11.7% vs 1.6%, *P* = 0.003), cryoprecipitate (16.7% vs 4.4%, *P* = 0.004) and mechanical ventilation (6.7% vs 0, *P* = 0.001) post NEC diagnosis. Furthermore, they were also in the a higher risk of blood transfusion prior to NEC diagnosis (16.7% vs 2.7%, *P* = 0.000), intravenous albumin (68.3% vs 29.7%, *P* = 0.000) or immunoglobulin (15.0% vs 3.8%, *P* = 0.007), anaerobic antimicrobial therapy (90.0% vs 62.6%, *P* = 0.000), vasoactive agents support (41.7% vs 17.0%, *P* = 0.000), and had a longer duration for broad spectrum antibiotics usage (*P* = 0.000), cessation of enteral feeding (*P* = 0.000), and gastrointestinal decompression (*P* = 0.000). Infants with CRP > 8 mg/L within 24 h of NEC diagnosis was significantly more in the group of infants with RBCT post NEC diagnosis than that in those without RBCT post NEC diagnosis and the lowest Hct post NEC diagnosis and prior NEC deterioration was significantly lower in the group of infants with RBCT post NEC diagnosis than that in those without RBCT post NEC diagnosis (Table 3).

**Table 3:** The treatment protocol and laboratory test of infants with or without red blood cell transfusion post diagnosis of NEC

Variables	Red Blood Cell Transfusion post NEC diagnosis		
	With (*n* = 60)	Without (*n* = 182)	*P*
Albumin transfusion, %(n)	68.3 (41)	29.7 (54)	0.000
Transfusion prior to NEC diagnosis, %(n)	16.7 (10)	2.7 (5)	0.000
Fresh frozen plasma transfusion post NEC diagnosis, %(n)	25.0 (15)	2.2 (4)	0.000
Platelet transfusion post NEC diagnosis, %(n)	11.7 (7)	1.6 (3)	0.003
Cryoprecipitate transfusion post NEC diagnosis, %(n)	16.7 (10)	4.4 (8)	0.004
Intravenous immunoglobulin, %(n)	15.0 (9)	3.8 (7)	0.007
Probiotics use, %(n)	48.3 (29)	50.5 (92)	0.766
Anaerobic antimicrobial therapy, %(n)	90.0 (54)	62.6 (114)	0.000
Vasoactive agents support, %(n)	41.7 (25)	17.0 (31)	0.000
Mechanical ventilation, %(n)	6.7(4)	0(0)	0.001
Broad spectrum antibiotics use [days, median (IQR)]	18 (12–24)	12 (9–16)	0.000
Cessation of enteral feeding [days, median (IQR)]	8.5 (6–13.75)	7 (4–9)	0.000
Gastrointestinal decompression [days, median (IQR)]	6 (1.25–9)	1 (0–4.25)	0.000
WBC count^a^ < 5 × 109/L or > 20 × 109/L, %(n)	20.0 (12)	15.9 (29)	0.467
Platelet count^a^ < 100 × 109/L, %(n)	11.7 (7)	7.7 (14)	0.343
CRP^a^ > 8 mg/L, %(n)	46.7 (28)	27.5 (50)	0.006
Lowest Hct ^b^ (%, mean ± SD)	25.8 ± 4.1	35.1 ± 6.4	0.000

^a^The baseline values were tested within 24 h of NEC diagnosis

^b^The Lowest Hct post NEC diagnosis and prior NEC deterioration

*WBC* White blood cell, *CRP* C-reactive protein, *NEC* Necrotizing enterocolitis, *Hct* Hematocrit, *SD* Standard deviation, *IQR* Interquartile range

### The influence of the RBCT post NEC diagnosis on the deterioration of NEC

The infants with RBCT post NEC diagnosis had the a significantly higher rate of NEC deteriorating from stage II to III (38.3% (23/60) vs 9.3% (17/182), *P* = 0.000) with comparison to those without RBCT post NEC diagnosis. By adjusting the propensity score, RBCT post NEC diagnosis was associated with an increased risk for NEC deteriorating from stage II to III (adjusted OR 6.06, 95%CI 2.94–12.50, *P* = 0.000).

## Discussion

RBCT is a common treatment to increase circulatory haemoglobin, improve tissue oxygenation, and maintain the stable of hemodynamics and the desired oxygenation [20]. Due to the iatrogenic blood loss, the small blood volume and immature hematopoietic system [21], newborn infants with severe complications received blood transfusion more frequently. It was reported that a transfusion rate of 13.4% in a population of patients, which included term neonates; 27.8% of the transfused patients were with a gestational age more than 34 weeks [22], 50 to 94% in infants with birth weight less than 1500 g and up to 95% in infants with birth weight less than 1000 g [23, 24]. Since 1980s, more and more evidences showed that RBCT played a controversial and doubtful role in the treatment of NEC [8–13, 25]. Some observational studies had reported that RBCT was associated with subsequent onset of NEC [8, 9]. However, no negative association between RBCT and the onset of NEC was proved in other studies [10, 11], even a protective effect was observed [12, 13]. Given that RBCT played a controversial and doubtful role in the onset of NEC, the neonatologist are facing an important dilemma in clinical practice. Firstly focusing on the relationship between RBCT post NEC diagnosis and deterioration of NEC on near-term and full term infants, the findings of the present study seem to indicate that RBCT post NEC diagnosis was associated with NEC deterioration from stage II to stage III.

Beena G and colleagues have found that infants were more likely to receive RBCT after diagnosed NEC than before, and approximately 90% NEC infants, with gestational age 23–32 weeks, received RBCT after diagnosed NEC [12]. Consistent with the previous study [12], infants were more likely to receive RBCT after diagnosis of NEC than before (24.8% versus 6.2%, *p* = 0.000) in the present study. However, the rates for RBCT after and before diagnosis of NEC in the present study are much lower than that in previous study (69% versus 90% after diagnosis and 6.2% versus 24.8% before diagnosis), and it can be explained by the higher gestational age and the larger birth weight of the near-term and full term infants in the present study.

Although a positive association between RBCT post NEC diagnosis and the deterioration of NEC was observed in this retrospective study, the underlying pathogenic mechanisms of this association were unknown. To some extent, the mechanisms which have been used to explain the transfusion associated NEC (TANEC) might be suitable for explaining the deterioration of NEC after transfusion. In most cases, when hemoglobin or hematocrit value fell below a certain threshold, a transfusion was invariably given to increase circulatory haemoglobin and improve tissue oxygenation. It was quite possible that infants with a history of RBCT post diagnosis of NEC were with a parallel diagnosis of severe anemia post diagnosis of NEC. And in our study, the lowest Hct value in the group of infants with RBCT post NEC diagnosis was significantly lower than that in those without RBCT post NEC diagnosis (Table 3). Could anemia per se be the reason for deterioration of NEC? Anemia can lead to poor perfusion and oxygen delivery to the intestine, resulting in a relative intestinal hypoxia and subsequent mucosal injury [20]. Animal anemia model had already shown that anemia could impair gut blood flow [26]. In anemia infants, intestinal injury was also detected prior to the RBCT [27], which established that infants with anemia already had harbored intestinal mucosal injury. What’s more, anemia could impair the normal maturation of vascular auto-regulation in the intestine, predisposing to intestinal ischemic injury [28, 29]. In murine model of anemia, inflammatory state in the intestinal mucosa with macrophage infiltration was found [30]. All these may possibly constitute an injury relevant to the deterioration of NEC, and anemia might be one possible mechanism for deterioration of NEC after RBCT.

Besides anemia, RBCT itself may also be the involved mechanism for deterioration of NEC. RBCT can alter blood flow to the bowel and augment intestinal injury theoretically [20]. During RBCT, there existed a sudden increasement of viscosity in the circulating blood, which had been proven to associate with the intestinal injury [20, 31]. And the affinity of RBCs for oxygen increases during storage, which shifts the recipient’s oxygen dissociation curve to the left, predisposing recipients to ischemia [32, 33]. What’s more, erythrocyte nitric oxide bioactivity losses rapidly during storage, which impairs the ability of RBCs to effect hypoxic vasodilation. Thus RBCs transfused to patients would act as a nitric oxide sink, resulting in vasoconstriction and ischemic insult to the intestine [34]. In principle, RBCTs are given to improve oxygen delivery to tissues, but, paradoxically, RBCTs fail to do it [32, 35], and may even make it worse [33, 36]. Another mechanism of TANEC, which centers on immunological injury, is also the plausible mechanism for intestinal injury post RBCT. Transfusion-related immunomodulation (TRIM) is speculated to be that RBCT trigger immune cell activation and related immunomodulation, whose likely manifestation is increasement of proinflamamatory cytokine production and endothelial activation [37]. In murine model of transfusion-associated NEC, MohanKumar et al. found that RBCT would activate intestinal mucosa cells, which were infiltrated by the macrophage, via a TLR4-mediated mechanism to cause bowel injury [30]. Keir et al. evaluated changes in circulating proinflammatory cytokine concentration and related downstream pathway in response to RBCT, assessed the potential proinflammatory effects for RBCs to be biologically active, and supported the existence of TRIM [38]. TRIM encompasses not only adverse proinflammatory and immunosuppressive responses but also the whole spectrum of posttransfusion effects on organs and tissues [37, 38], which may partly explain the association between RBCT and the pathogenesis of several high neonatal morbidities affecting the brain [39–41], eyes [40, 41], lung [41, 42], and gut [40, 41]. Some other studies had also confirmed that proinflammatory cytokine concentration increased in infants who underwent a RBCT [21, 43]. Cytokines have been confirmed to play an important role in mediating intestinal inflammation and injury in the pathogenesis of NEC [44, 45]. TRIM is the mechanism to explain how RBCT can induce an increase in cytokine serum level and to partly explain the association between RBCT and the deterioration of NEC. Although we regarded the foregoing as different possible pathogenic mechanisms, they might not be mutually exclusive; conceivably, elements of these mechanisms could operate at the same time.

There were some limitations existing in the present study, such as the inherent errors and bias of retrospective studies. This retrospective cohort study was also limited by its cohort design, failure to take some unreported clinical risk factors for NEC and/or exposures to RBCTs into consideration, and there still existed some unmeasured confounders which might affect the propensity score. In order to minimize ascertainment bias, we identified only stage II NEC cases in our hospital databases, and excluded all suspected NEC cases, resulting in a relatively small number of NEC cases included in this study. Because of the small sample, we did not further divide the included cases into the subgroups of Bell’s stage. We were unable to define the precise time of onset and diagnosis of NEC. The time of onset of NEC, defined as the day of the first presentation of non-specific signs and symptoms, and the time of diagnosis of NEC, defined as the day of the abdominal X-ray or the ultrasound results fit the diagnostic criteria for stage II NEC, may be made several hours after NEC occured and confirmed. Absence of a standardized feeding protocol during the study period may have influenced the results of our study [46, 47]. As no breast milk bank in our hospital, the formula was used for all infants during hospitalization. And lack of early colostrums feeding would contribute to the deterioration of NEC [4], we did not know whether formula feeding could have an influence on the deterioration of NEC in our study or not. Most importantly, in this study, the association between RBCT post NEC diagnosis and the deterioration of NEC, even with a statistically significant difference, does not prove a cause and effect relationship. Thus, the antecedent RBCT might have no pathogenic role on the deterioration of confirmed NEC. Rather, the RBCT might be an epiphenomenologic marker of deterioration of NEC. In order to determine whether RBCT cause the deterioration of NEC will require a different experimental approach.

In conclusion, NEC infants who required RBCT post NEC diagnosis were more likely to associate with the deterioration from stage II to III in full-term and near-term infants.

## Supplementary Information

**Additional file 1.**

## Data Availability

The datasets used and analysed during the current study are available from the corresponding author on reasonable request.

## Abbreviations

NEC: Necrotizing enterocolitis
RBCT: Red blood cell transfusion
NICU: Neonatal intensive care unit
HIS: Hospital information system
CRP: C-reactive protein
Hct: Hematocrit
OR: Odds ratio
CI: Confidential interval
NICU: Neonatal intensive care units
TANEC: Transfusion associated NEC
TRIM: Transfusion-related immunomodulation
