# Supplementary material for: Red blood cell transfusions post diagnosis of necrotizing enterocolitis and the deterioration of necrotizing enterocolitis in full-term and near-term infants: a propensity score adjustment retrospective cohort study
Source: BMC Pediatr. 2022 Apr 15;22:211. doi: 10.1186/s12887-022-03276-4 (PMC9012001; doi:10.1186/s12887-022-03276-4)
Supplement: Supplementary file 1 — Additional file 1. [file 12887_2022_3276_MOESM1_ESM.docx]

| Guidelines for transfusion of RBCs in patients less than four months of age |
| --- |
| 1. Hct <20% with low reticulocyte count and symptoms of anemia*  2. Hct <30% with an infant:  ● On <35% hood O_2_  ● On O_2_ by nasal cannula  ● On continuous positive airway pressure and/or intermittent mandatory ventilation with mechanical ventilation with mean airway pressure <6 cm H_2_O  ● With significant apnea or bradycardia†  ● With significant tachycardia or tachypnea‡  ● With low weight gain§  3. Hct <35% with an infant:  ● On >35% hood O_2_  ● On continuous positive airway pressure/intermittent mandatory ventilation with mean airway pressure 6-8 cm H_2_O  4. Hct <45% with an infant:  ● On ECMO  ● With congenital cyanotic heart disease |
| * Tachycardia, tachypnea, poor feeding.  † More than six episodes in 12 hr or two episodes in 24 hr requiring bag and mask ventilation while receiving therapeutic doses of methylxanthines.  ‡ Heart rate >180 beats/min for 24 hr; respiratory rate >80 breaths/min for 24 hr.  § Gain of <10 g/day observed over 4 days while receiving 100 kcal/kg/day |
